# Predictors of chronic breathlessness: a large population study

**DOI:** 10.1186/1471-2458-11-33

**Published:** 2011-01-12

**Authors:** Jacqueline A Bowden, Timothy HM To, Amy P Abernethy, David C Currow

**Affiliations:** 1 Cancer Council, South Australia, 202 Greenhill Rd, Parkside, South Australia, Australia; 2 Southern Adelaide Palliative Services, Repatriation General Hospital, 700 Goodwood Rd, Daw Park, South Australia, 5041, Australia; 3 Discipline of Palliative and Supportive Services, Flinders University, Sturt Road, Bedford Park, South Australia, 5042, Australia; 4 Division of Medical Oncology, Department of Medicine, Duke University Medical Centre, Durham, North Carolina, 27710, USA; 5 Centre for Clinical Change, School of Medicine, Flinders University, Sturt Road, Bedford Park, South Australia,5042, Australia

## Abstract

**Background:**

Breathlessness causes significant burden in our community but the underlying socio-demographic and lifestyle factors that may influence it are not well quantified. This study aims to define these predictors of chronic breathlessness at a population level.

**Methods:**

Data were collected from adult South Australians in 2007 and 2008 (n = 5331) as part of a face-to-face, cross-sectional, whole-of-population, multi-stage, systematic area sampling population health survey. The main outcome variable was breathlessness in logistic regression models. Lifestyle factors examined included smoking history, smoke-free housing, level of physical activity and body mass index (obesity).

**Results:**

The participation rate was 64.1%, and 11.1% of individuals (15.0% if aged ≥50 years) chronically had breathlessness that limited exertion. Significant bivariate associations with chronic breathlessness for the whole population and only those ≥50 included: increasing age; female gender; being separated/divorced/widowed; social disadvantage; smoking status; those without a smoke-free home; low levels of physical activity; and obesity. In multi-variate analyses adjusted for age, marital status (p < 0.001), physical activity (p < 0.001), obesity (p < 0.001), gender (p < 0.05) and social disadvantage (p < 0.05) remained significant factors. Smoking history was *not *a significant contributor to the model.

**Conclusions:**

There is potential benefit in addressing reversible lifestyle causes of breathlessness including high body mass index (obesity) and low levels of physical activity in order to decrease the prevalence of chronic breathlessness. Clinical intervention studies for chronic breathlessness should consider stratification by body mass index.

## Background

Chronic breathlessness causes a significant burden in our community for the individuals with the symptom, their carers and the health system. Many presentations in primary care and to the emergency departments of hospitals are driven primarily by acute breathlessness or acute worsening of long-term breathlessness for several prevalent conditions such as chronic obstructive pulmonary disease, cardiac failure, acute coronary syndrome, and asthma[1].

Breathlessness is a complex sensation with a wide range of factors that can generate and sustain it. The efferent drive to breathe is mediated through the phrenic nerves and nerves supplying the intercostal muscles. When these pathways are stimulated out of proportion to their ability to respond by afferent signals from chemo- and mechano-receptors, the mismatch between the two systems generates the feeling of breathlessness[2]. Central emotional factors such as anxiety, fear or anger can also contribute to breathlessness. Further, breathlessness can be divided into at least two components with different trajectories as the sensation worsens - the intensity of breathlessness and the degree to which it is perceived to be unpleasant[3].

The management of chronic breathlessness at a population level requires three parallel approaches: reducing or modifying lifestyle factors that cause breathlessness; remedying any reversible causes that may contribute to, or exacerbate breathlessness; and ensuring that any remaining breathlessness is adequately palliated.

The prevalence and the intensity of chronic breathlessness experienced in the community, irrespective of health service utilisation, has previously been reported[4,5]. Recently, a unique dataset has been created by joining databases from different research organisations (Flinders University and Cancer Council South Australia), who each commissioned questions in the same years of the South Australian Health Omnibus Survey. This allowed population data on chronic breathlessness to be linked to key lifestyle factors that may contribute to breathlessness, including tobacco smoking, body mass index (BMI), physical activity, and the presence of a smoker in the household. The aim of this study was to determine the impact of known socio-demographic and lifestyle factors at a population level that may influence the prevalence of chronic breathlessness. The null hypothesis was that there were no factors that predict chronic breathlessness at a population level.

## Methods

### Setting

South Australia has a population of 1.6 million people (7% of the Australian population), most of whom live in one metropolitan centre, Adelaide (1.1 million people). The remaining population live in small regional centres (maximum population in any other centre: <30,000).

### Survey tool

The Health Omnibus Survey is an annual, face-to-face, cross-sectional, whole-of-population multi-stage, systematic area sampling survey where respondents are interviewed in their own homes. It is run for health and research organisations and the methodology has already been described in detail[5]. Data were collected as part of the South Australian Health Omnibus Survey in 2007 and 2008 from approximately 3000 South Australians adults annually. Data were directly weighted to the South Australian population by age, gender and geographic area (urban or rural).

### Questions in the Health Omnibus survey

More than 200 questions about health and social issues (spanning medication use to childcare, arthritis to exercise habits) are included annually in interviews of 60 to 90 minutes duration. The only standard questions are the demographic factors which are provided back to all subscribers. All other questions are generated by researchers and health services on a user-pays system for the 'space' for a question, the answers to which are only supplied to the subscribing researcher.

Prior to the main survey, a pilot study of 50 interviews with members of the general public is conducted annually to test questions, validate the survey instrument and reassess survey procedures.

### Sampling schema

**Figure 1:**
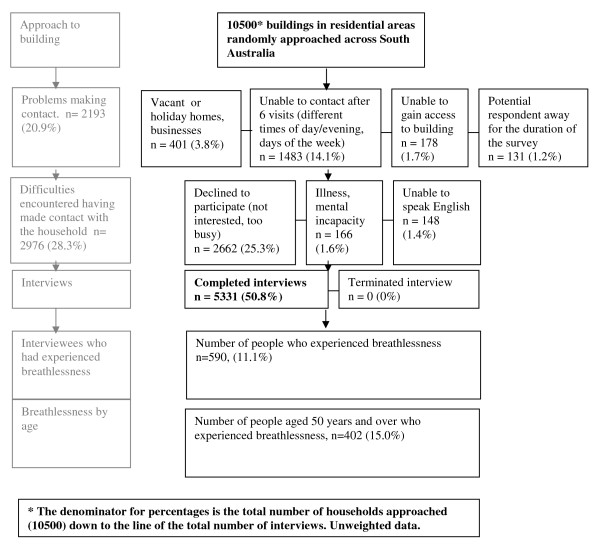
**Flowchart of participation in the South Australian Health Omnibus 2007 and 2008 Participation rate 64.1% (5331/(10500-2193))**.

The survey is carried out between September and December. Each year, more than 5000 properties are approached seeking a resident to participate in the interview (Figure 1). 'Properties' include vacant land, or businesses (retail, light industrial or warehouses) rather than just residences. Therefore participation rates are calculated on the number of relevant potential participants with whom contact is actually made, not the number of properties approached.

In Australia, the smallest administrative data area is a census collector district (CD) which has approximately 200 residences. There are 2041 CDs in Adelaide of which 340 were randomly selected annually, each with an equal chance of selection. In non-metropolitan areas, there are 1010 CDs from which 100 are chosen. The eight towns in the state with more than 10,000 people were included and towns with populations of 1,000 to 10,000 were randomly selected in proportion to the size of the town. Starting points within each CD were randomly selected using a map with numbered corners and a random number generator. Each collector then used a set algorithm to move from the starting point using a skip pattern that selected every fourth property in each cluster.

### Analysis

Levels of breathlessness were ascertained using the modified Medical Research Council (mMRC) scale[6,7]. The question asked 'thinking back over the last 6 months, have you had an episode of breathlessness that has lasted more than 3 months'. Response categories were: 0 = None; 1 = 'I get short of breath when hurrying on the level or up a slight hill'; 2 = 'I have to stop for breath when walking at my own pace on the level'; 3 = 'I stop for breath after walking 100 meters or after a few minutes on the level'; or 4 = 'I am too breathless to leave the house'. [Table 1] This question allows respondents to categorise the level at which their exertion is limited by breathlessness. This has previously been validated in a range of populations with known respiratory pathology. For analysis, breathlessness was divided by mMRC into 'none' or all others (1, 2, 3 and 4). Additional analyses were undertaken in three categories: no breathlessness; grade 1 mMRC breathlessness and categories 2, 3 and 4.

**Table 1:** Demographics and responses to key variables of a population survey of 5331 people in South Australia in the 2 years 2007-8.

Variable	Levels	Total population % (n = 5331)	50 years and over % (n = 2685)
Gender	Male	42.3	40.7
	Female	57.7	59.3
Marital status	Married	47.6	54.6
	De facto	8.7	3.7
	Separated/divorced	13.5	16.9
	Widowed	9.9	19.2
	Never married	20.0	5.3
	Not stated	0.3	0.3
Age	<35	24.3	-
	35-49	25.3	-
	50-64	25.8	51.3
	>65	24.5	48.7
Level of Disadvantage	Most disadvantaged	22.5	22.7
	2	21.9	21.5
	3	17.9	17.9
	4	17.2	16.8
	Least disadvantaged	20.5	21.1
Breathlessness	None	88.9	85.0
	Breathless when hurrying on level or up a slight hill	7.5	9.2
	Stop for breath when walking up at my own pace on the level	1.7	2.9
	Stop for breath after 100 m or after a few minutes on the level	1.5	2.3
	Too breathless to leave the house	0.4	0.6
	Not stated	<0.1	<0.1
Smoking history	Never smoker	50.1	49.6
	Ex-smoker	30.4	38.3
	Current smoker	19.5	12.1
Smoke-free home	Yes	85.6	87.1
	No	14.4	12.9
Physical activity	No activity	20.2	25.1
	Some activity but not sufficient	41.4	42.1
	Sufficient physical activity	38.4	32.9
Body mass index	Under weight	2.6	1.5
	Healthy weight	41.0	36.1
	Overweight	33.5	37.2
	Obese	23.0	25.2

# Weighted to the South Australian population

Age was included in the single-variate analysis for all participants as a categorical variable (given the different potential causes of chronic breathlessness by age group - asthma in the young, chronic obstructive pulmonary disease in the elderly, obesity across the lifespan) and in the multi-variate analysis as a continuous variable. Postcode data were merged with the Socio-economic Index for Areas 2001 - Index of Disadvantage (SEIFA) to allow analysis by postcode-specific ecological measures of disadvantage that integrate variables including income, educational attainment, levels of employment and how skilled the workforce is in that particular geographic area[8].

Smoking status was determined by asking respondents whether they currently smoke cigarettes, cigars, pipes or other tobacco products on a 'daily', 'at least weekly', 'less often than weekly' basis or 'not at all'. An associated variable was whether there was currently a smoker in the household. The questions on smoking and lifestyle were in a separate part of the survey to the question on breathlessness.

National physical activity guidelines for Australians recommend 30 minutes of moderate intensity physical activity on most, preferably all, days of the week. Individuals that undertook 150 minutes or more per week on 5 or more occasions were classified as undertaking 'sufficient physical activity' for general health benefit. Individuals that undertook physical activity but less than a total of 150 minutes or less than 5 occasions per week were classified as 'some activity but not sufficient'.

Body mass index (BMI) was calculated from weight in kilograms divided by height in metres squared. A healthy index is considered to be between 18.5 and 25. Underweight is defined as a BMI of less than 18.5, overweight is defined as a BMI between 25 and 30 and obesity as over 30[9]. There are strong correlations between BMI and a number of chronic diseases[10]. A number of participants (n = 708) could not recall either their height, or their weight to allow BMI calculations to be undertaken. These people were therefore not represented in the logistic regression models.

Given the cumulative effects of lifestyle factors such as smoking, physical activity or body mass potentially have on breathlessness, a sub-group analysis was undertaken for people aged 50 years and over to determine the later effects of these factors on chronic breathlessness.

### Statistical Analysis

Data for 2007-2008 were merged. Statistical analyses were undertaken using StataSE 10.0 [StataCorp, College Station, Texas 2007], the estimating tools of which account for clustered and stratified survey designs. Logistic regression modelling was undertaken and WALD tests were calculated to determine whether variables warranted inclusion in the multi-variate logistic regressions at p < 0.05[11].

### Power calculation

Based on the previous population study, it was anticipated that the prevalence of breathlessness would be 8%[5]. A sample size of 4,403 would allow the estimation of this proportion with a 95% confidence interval of ±0.08%. Allowing for a 60% participation rate at least 7,340 people needed to be approached.

### Ethics and consent

The survey received South Australian Department of Health Research Ethics Committee approval. Given that this was a community survey of people in their own homes, verbal consent from participants was acceptable before interview, and continued participation accepted as continuing consent.

### Study reporting

This paper complies with the STROBE consensus statement on reporting observational studies[12].

## Results

Over the two years included in this analysis, a total of 5331 people responded. The participation rate (calculated as the number of respondents as a fraction of the number of people who were contacted) was 64.1%. Responses to specific questions included in these analyses are outlined in Table 1.

Single-variate analysis of demographic information revealed that people who could not provide data for calculation of body mass index (n = 708) were more likely to be female, less likely to be married and more likely to be disadvantaged than those who recalled their height and weight.

### Predictors of breathlessness

Predictors of dyspnoea were explored in bivariate and multivariate analyses for all respondents and separately for people aged 50 years and over.

In direct comparisons between breathlessness and each of the demographic and lifestyle factors available for analyses, factors showed a gradient towards increasing breathlessness across the continuum for age, marital status, socio-economic disadvantage, smoking history, smoke-free home, physical activity and obesity. [Table 2] In multi-variate analyses adjusted for age, gender, marital status and social disadvantage, physical activity and obesity remained significant factors. Smoking history and a smoke-free home were no longer significant contributors to this model. [Table 3] The Archer-Lemeshow F-adjusted mean residual goodness-of-fit test confirmed adequacy of the model (P = 0.99). Further univariate analysis by grade of dyspnoea revealed that people with moderate breathlessness were more likely to be female than those with no or severe breathlessness (χ^2 ^= 22.4, df = 2, p < 0.001). Those with severe breathlessness were less likely to be married and more likely to be separated or widowed (χ^2 ^= 92.8, df = 10, p < 0.001) and older than other groups (χ^2 ^= 154.8, df = 6, p < 0.001). Those with severe breathlessness were also more likely to live in disadvantaged areas (χ^2 ^= 60.2, df = 8, p < 0.001) and were more likely to be ex-smokers than the other groups (χ^2 ^= 27.7, df = 4, p < 0.001) There was a clear gradient by grade of breathlessness whereby as severity of breathlessness increased, BMI increased (χ^2 ^= 75.8, df = 6, p < 0.001) and physical activity decreased (χ^2 ^= 157.1, df = 4, p < 0.001).

**Table 2:** Single-variate analysis for factors associated with prevalence of breathlessness for 2007 and 2008 in a face-to-face population survey in South Australia

Variable	Levels	n	Odds ratio	95% CI	p value
Gender	Male	2257	1		
	Female	3074	1.52	1.21-1.90	<.001
Marital status	Married	2539	1		
	De facto	464	0.67	0.45-0.10	<.05
	Separated/divorced	722	1.73	1.21-2.48	<.01
	Widowed	526	2.67	2.03-3.51	<.001
	Never married	1066	0.78	0.58-1.05	.10
	Not stated	14	0.56	0.07-4.39	.58
Age	<35	1296	1		
	35-49	1350	1.00	0.71-1.40	1.0
	50-64	1377	1.72	1.28-2.32	<.001
	>65	1308	3.13	2.34-4.20	<.001
Level of Disadvantage	Most disadvantaged	1198	1		
	2	1167	0.63	0.45-0.87	<.01
	3	954	0.63	0.45-0.89	<.01
	4	918	0.60	0.44-0.83	<.01
	Least disadvantaged	1094	0.46	0.33-0.65	<.001
Smoking history	Never smoker	2672	1		
	Ex-smoker	1622	1.49	1.18-1.88	<.01
	Current smoker	1037	1.34	1.01-1.77	<.05
Smoke-free home	Yes	4472	1		
	No	751	1.53	1.19-1.97	<.05
Physical activity	No activity	1073	1		
	Some activity but not sufficient	2195	0.56	0.43-0.73	<.001
	Sufficient physical activity	2034	0.34	0.25-0.46	<.001
Body mass index*	Healthy weight	1894	1		
	Overweight	1547	1.14	0.86-1.52	.37
	Obese	1063	2.49	1.81-3.43	<.001
	Underweight	119	1.14	0.56-2.32	.71

# Single-variate logistic regression with breathlessness as the dependent variable. Controlling for clustering and weighted to the South Australian population

**Table 3:** Multi-variate analysis for factors associated with prevalence of breathlessness for 2007 and 2008 in a face-to-face population survey

Variable*	Levels	Odds ratio	95% CI	p value
Gender	Male	1		
P < 0.05	Female	1.40	1.07-1.82	<.05
Marital status**	Married	1		
P < 0.001	De facto	0.92	0.59-1.45	.72
	Separated/divorced	1.87	1.28-2.75	<.01
	Widowed	1.56	1.10-2.20	<.05
	Never married	1.81	1.27-2.59	<.01
Level of Disadvantage	Most disadvantaged	1		
P < 0.05	2	0.66	0.47-0.93	<.05
	3	0.62	0.43-0.89	<.01
	4	0.68	0.48-0.97	<.05
	Least disadvantaged	0.55	0.38-0.80	<.01
Physical activity	No activity	1		
P < 0.001	Some activity but not sufficient	0.60	0.44-0.81	<.01
	Sufficient physical activity	0.42	0.30-0.59	<.001
Body mass index	Healthy weight	1		
P < 0.001	Overweight	1.07	0.80-1.43	.66
	Obese	2.13	1.53-2.96	<.001
	Underweight	1.15	0.57-2.29	0.70

*Other variables included in the analysis were as follows: smoke-free home (p = 0.17), smoking history (p = 0.13)

# Multi-variate logistic regression with breathlessness as the dependent variable. Controlling for age, clustering and weighted to the South Australian population

** unstated marital status dropped due to collinearity

When exploring the subset of people in the cross-section over the age of 50, the results looked similar. Lack of physical activity, socio-economic disadvantage, being widowed or divorced/separated and age remained factors of interest, as did obesity and interestingly, those categorised as underweight. [Table 4] Adjusting for age, in multi-variate analysis, obesity, lack of physical activity, and social disadvantage remained significant predictors of breathlessness across the community. [Table 5] The predictive value of marital status and being underweight was not significant, and again the predictive value of smoking history or smoke exposure in the home was not evident in the multivariate analysis. The Archer-Lemeshow F-adjusted mean residual goodness-of-fit test confirmed adequacy of the model (P = 0.92). Interaction terms were also investigated but were not found to add to model fit or suggest heterogeneity of effects across other predictors.

**Table 4:** Single-variate analysis for factors associated with prevalence of breathlessness from 2007 and 2008, for individuals aged 50+ in a face-to-face population survey in South Australia

Variable	Levels	n	Odds ratio	95% CI	p value
Gender	Male	1092	1		
	Female	1593	1.23	0.94-1.61	.12
Marital status	Married	1466	1		
	De facto	99	1.17	0.67-2.06	.58
	Separated/divorced	453	1.54	0.98-2.42	.06
	Widowed	516	2.04	1.52-2.74	<.001
	Never married	142	1.28	0.76-2.16	.35
	Not stated	9	0.80	0.10-6.67	.84
Age	50-64	1377	1		
	>65	1308	1.82	1.45-2.30	<.001
Level of Disadvantage	Most disadvantaged	609	1		
	2	578	0.60	0.41-0.87	<.01
	3	481	0.69	0.45-1.05	.08
	4	450	0.65	0.44-0.97	<.05
	Least disadvantaged	567	0.41	0.27-0.63	<.001
Smoking history	Never smoker	1331	1		
	Ex-smoker	1028	1.25	0.96-1.63	.10
	Current smoker	326	1.28	0.89-1.85	.19
Smoke-free home	Yes	2290	1		
	No	338	1.29	0.93-1.81	.13
Physical activity	No activity	669	1		
	Some activity but not sufficient	1123	0.57	0.42-0.78	<.001
	Sufficient physical activity	878	0.29	0.21-0.42	<.001
Body mass index	Healthy weight	836	1		
	Overweight	863	0.89	0.62-1.29	.54
	Obese	585	1.78	1.21-2.64	<.01
	Underweight	34	3.17	1.34-7.49	<.01

# Single-variate logistic regression with breathlessness as the dependent variable. Controlling for clustering and weighted to the South Australian population

**Table 5:** Multi-variate analysis for factors associated with prevalence of breathlessness for 2007 and 2008 for individuals aged 50+ in a face-to-face population survey

Variable*	Levels	Odds ratio	95% CI	p value
Level of Disadvantage	Most disadvantaged	1		
P < 0.05	2	0.62	0.41-0.96	<.05
	3	0.61	0.39-0.96	<.05
	4	0.72	0.46-1.13	.15
	Least disadvantaged	0.48	0.30-0.77	<.05
Physical activity	No activity	1		
P < 0.001	Some activity but not sufficient	0.61	0.42-0.90	<.05
	Sufficient physical activity	0.36	0.22-0.57	<.001
Body mass index*	Healthy weight	1		
P < 0.001	Overweight	0.85	0.57-1.27	.52
	Obese	1.81	1.08-2.78	<.05
	Underweight	1.86	0.75-4.61	.18

*Other variables included in the analysis were as follows: gender (p = 0.10), marital status (p = 0.10), smoke-free home (p = 0.94), smoking history (p = 0.18)

# Multi-variate logistic regression with breathlessness as the dependent variable. Controlling for age, clustering and weighted to the South Australian population

** unstated marital status dropped due to collinearity

In adults who were classified as obese (n = 1063), 18.1% experienced breathlessness compared to 8.7% of those who were not obese. Among adults aged 50 years and over, breathlessness was experienced by 22.2% of those who were obese, compared to 12.3% who were not obese.

### Sensitivity analysis

The direction and magnitude of findings were confirmed by conducting the same analysis without population weighting.

## Discussion

Chronic breathlessness is a prevalent problem in the community. A relationship between breathlessness, reduced activity and obesity has been described in a study of older adults randomly selected from the community[13]. However this relationship has not been documented at a broader population level prior to this study, independent of health service presentations or selection of obese subjects in the first place. This study also suggested that socio-economic disadvantage is associated with higher levels of moderate to severe chronic breathlessness in our community.

Given the increasing prevalence of obesity and sedentary lifestyles with lack of physical exercise in the community, these findings are a cause for concern[14]. For most people, these factors are modifiable and there is an important public health message that is not limited to mortality - lower levels of physical activity and increasing weight are likely to increase the risk of chronic breathlessness at all ages, potentially creating and perpetuating a vicious cycle of decline. People living in lower socio-economic areas may benefit from interventions to reduce their weight and increase their physical activity. Given the levels of severe and extreme breathlessness associated with obesity in these current data, even the most basic activities of daily living or social interactions are potentially restricted for people with severe obesity.

The World Health Organisation indicated that in 2005, at least 400 million adults world-wide were obese and projections indicate that by 2015 approximately 700 million adults will be obese[9]. Extrapolating the findings of this study (not accounting for factors that may influence the generalisability of these findings to some countries) estimations indicate that of these people, 130 million adults worldwide may experience chronic breathlessness associated with obesity, placing a significant burden on society and health systems. In terms of mortality, recent estimates suggest that a two point rise in the population's BMI has the same effect on population-based all cause mortality as a 10% increase in smoking prevalence [15] although there are complex interactions between the two factors.

Higher rates of dyspnoea have been associated with obesity in people over the age of 50[13], although the reported effects of obesity have not always been consistent with worsening dyspnoea[16-18]. Putative mechanisms for breathlessness in the obese include reduced end expiratory lung volume[19], reduced total lung capacity[19], increased work of breathing at a given level of exertion[20], decreased forced expiratory flow, decreased maximum voluntary ventilation, systemic hypoxia worsening metabolic function globally [21] and increased airway reactivity causing bronchoconstriction even in non-asthmatics. Conversely, in people who have had morbid obesity reversed by bariatric surgery, vital capacity and expiratory reserve volume increased after weight loss[22]. Given the differences in sampling in the reported papers to date, it is timely to have population-based data available of breathlessness cross-tabulated with in the community.

Of note, tobacco smoking was not significant in the final multivariate analysis for the whole population, nor for the sub-group of people over 50 years of age in the causes of breathlessness controlling for other available factors. This may reflect the relative decrease in the prevalence of adult tobacco smokers compared to the rapidly increasing adult rates of obesity in our society. Another factor that may influence such differences in prevalence is that obesity may cause chronic breathlessness from the time that obesity develops while smoking may have a significant lead time before breathlessness manifests itself chronically. The major symptomatology of breathlessness from smoking is in the last decade of a person's life. It may be that with increasing age and accumulated chronic disease and comorbidities, breathlessness is more likely to be multi-factorial rather than due simply to smoking itself. For instance, cardiac or respiratory dysfunction, deconditioning, and frailty are more prevalent in this population and may contribute to breathlessness independently of smoke exposure.

### Limitations - methodology

There is obviously the risk of highly correlated factors being used in the analysis. Smoking rates are inversely proportional to social disadvantage, household income and lower levels of education. This is accounted for in the multivariate analyses.

The study relies on self-report but, importantly, there is no particular secondary gain or loss in responding honestly to an interviewer who is unlikely to be encountered again. Importantly, this interviewer is not a health professional with whom the respondent is in a dependent relationship. Such deficits in self-reporting may include the data to calculate BMI, where there were missing data for individuals possibly under-representing levels of obesity in the community and hence the association with breathlessness.

This study sought to define associations with breathlessness from socio-demographic factors and lifestyle factors. Findings would be strengthened in the future if underlying clinical pathologies were included.

### Limitations - sample

This study may not represent the full effects of tobacco smoke in the community as respondents had to be more than 15 years of age. There are compelling data that passive exposure to tobacco smoke is associated with higher levels of respiratory problems in infants and children[23]. Given rising rates of childhood obesity and increasingly sedentary behaviour in our community, the data also do not reflect the impact of chronic breathlessness for those under 15 years of age[10,24].

### Implications for research

For future population studies, work exploring the attributable contribution of different underlying pathologies (heart disease, respiratory disease, neuromuscular dysfunction) would further enhance the work.

In research design, it could be argued that future clinical studies on evaluating interventions for the symptomatic treatment of breathlessness should consider stratifying by body mass index (BMI) given the findings in this study.

Some questions arise directly from this study that need other methodologies to answer. Given the evidence of improvement in respiratory function and dyspnoea following successful weight loss after bariatric surgery [22,25], does weight loss without surgical intervention also reduce breathlessness? The relationship to define causality (obesity worsening breathlessness; breathlessness on exertion worsening obesity; or both) rather than simply an association needs to be elucidated in future work. Does gentle exercise help to reduce overall breathlessness, and potentially start to reverse some key metabolic dysfunction caused by obesity?

### Implications for practice

There is a fundamental challenge in asking people to make changes to their lifestyles now (smoking cessation, increased physical activity) in order to improve likely future health states (avoiding chronic breathlessness). It is not clear from these data whether reduction in weight or increase in physical activity would reduce breathlessness.

In a person presenting with breathlessness, not only will there continue to be the need to look for system-specific reversible pathology (chronic arrhythmias, chronic obstructive pulmonary disease, interstitial lung disease) but to proactively address reversible lifestyle and medical causes for high BMI and encourage increased level of physical activity. Opportunistically in clinical encounters, anything that can contribute to the reversal of the cycle of obesity/breathlessness/obesity, particularly among people living in lower socio-economic areas is to be encouraged.

Even when underlying causes of breathlessness have been optimally treated, there will remain a large cohort in the community with chronic breathlessness. Whilst there are interventions being identified that can reduce breathlessness without otherwise compromising the health of the person, it is imperative that whole of population estimates are available to inform planners and clinicians about the number of people who are likely to require symptomatic treatment[2,26-28].

## Conclusion

Given the rising prevalence of people who are overweight or obese and the decreasing levels of physical activity in the community, the association with chronic breathlessness is striking. The absence of tobacco smoke as a contributing factor is surprising but may reflect the reducing rates of tobacco use in the community. At a public health level, the need to manage modifiable lifestyle factors to optimise people's wellbeing continues to be imperative.

## Competing interests

The authors declare that they have no competing interests.

## Authors' contributions

Conception and design - DC, AA, JB; acquisition of data - DC, AA, JB; analysis and interpretation of data - DC, AA, TT, JB; drafting of the manuscript - JB, DC; critical revision of the manuscript for important intellectual content - AA, TT, JB, DC; read and approved the final manuscript - DC, AA, JB, TT; statistical analysis - JB; obtaining funding - DC, AA, JB; read and approved final manuscript - DC, AA, JB, TT.

## Pre-publication history

The pre-publication history for this paper can be accessed here:

http://www.biomedcentral.com/1471-2458/11/33/prepub
